# Healthcare resource utilization of patients with mitochondrial disease in an outpatient hospital setting

**DOI:** 10.1186/s13023-023-02746-x

**Published:** 2023-05-29

**Authors:** Sameen Haque, Karen Crawley, Rupendra Shrestha, Deborah Schofield, Carolyn M. Sue

**Affiliations:** 1 Nepean Hospital, Derby Street, Kingswood, NSW 2747 Australia; 2 The Kolling Institute, Royal North Shore Hospital, Reserve Road, St Leonards, NSW 2065 Australia; 3 Centre for Economic Impacts of Genomic Medicine (GenIMPACT), Macquarie Business School, Macquarie University, Eastern Rd, Macquarie Park, NSW 2109 Australia; 4 Neuroscience Research Australia, University of New South Wales, Sydney, Australia

**Keywords:** Mitochondrial diseases, Mitochondrial disorders, Health care costs, Health services, Health resources, Outpatients

## Abstract

**Background and objectives:**

Mitochondrial diseases present as multi-system disorders requiring a comprehensive multidisciplinary approach. The data on healthcare resource utilization associated with mitochondrial diseases and the clinical drivers of these costs are limited including for the out-patient setting where the majority of the clinical care for mitochondrial disease patients occurs. We performed a cross-sectional retrospective study of out-patient healthcare resource utilization and costs for patients with a confirmed diagnosis of mitochondrial disease.

**Methods:**

We recruited participants from the Mitochondrial Disease Clinic in Sydney and stratified them into three groups: those with mitochondrial DNA (mtDNA) mutations (Group 1), those with nuclear DNA (nDNA) mutations and the predominant phenotype of chronic progressive external ophthalmoplegia (CPEO) or optic atrophy (Group 2) and those without a confirmed genetic diagnosis but clinical criteria and muscle biopsy findings supportive of a diagnosis of mitochondrial disease (Group 3). Data was collected through retrospective chart review and out-patient costs were calculated using the Medicare Benefits Schedule.

**Results:**

We analyzed the data from 91 participants and found that Group 1 had the greatest average out-patient costs per person per annum ($838.02; SD 809.72). Neurological investigations were the largest driver of outpatient healthcare costs in all groups (average costs per person per annum:—Group 1: $364.11; SD 340.93, Group 2: $247.83; SD 113.86 and Group 3: $239.57; SD 145.69) consistent with the high frequency (94.5%) of neurological symptoms. Gastroenterological and cardiac-related out-patient costs were also major contributors to out-patient healthcare resource utilization in Groups 1 and 3. In Group 2, ophthalmology was the second-most resource intensive specialty ($136.85; SD 173.35). The Group 3 had the greatest average healthcare resource utilization per person over the entire duration of out-patient clinic care ($5815.86; SD 3520.40) most likely due to the lack of a molecular diagnosis and a less customized management approach.

**Conclusion:**

The drivers of healthcare resource utilization are dependent on the phenotype–genotype characteristics. Neurological, cardiac, and gastroenterological costs were the top three drivers in the out-patient clinics unless the patient had nDNA mutations with predominant phenotype of CPEO and/or optic atrophy wherein ophthalmological-related costs were the second most resource intensive driver.

**Supplementary Information:**

The online version contains supplementary material available at 10.1186/s13023-023-02746-x.

## Background

Mitochondrial diseases comprise a heterogenous group of genetic disorders resulting from mutations in either mitochondrial DNA (mtDNA) or nuclear DNA (nDNA) [1].

Clinical manifestations are protean [2–4] and the spectrum of disease severity ranges from mild or oligosymptomatic disease to severe or life-threatening multi-organ involvement. The multi-systemic manifestations may include (but are not limited to) myopathy, seizures, strokes, visual impairment, diabetes, sensorineural hearing loss, cardiomyopathy and gastrointestinal dysmotility [5–7].

Patients with mitochondrial disease require a comprehensive approach utilizing both in-patient and out-patient multidisciplinary care models with input from multiple medical specialties and allied health divisions [8–12]. Mitochondrial diseases, being representative of progressive, multisystemic chronic conditions, demonstrate the increasing challenge and impact these and similar illnesses have on population health models, not just within Australia but worldwide. As a group, chronic conditions have become “the leading cause of illness, disability and death in Australia” [13], continually adding not just to premature mortality, but also disability rates [14]. However, the persistence and practice of acute care models within public health care systems, is ineffective as costs spiral upwards without the desired improvements [15].

By better understanding healthcare resource utilisation and related costs within a chronic condition such as mitochondrial disease; future practice, skills, prevention, resources, streamlining of services, and disease monitoring systems (both within symptomatic and asymptomatic patients) to predict and prevent complications, will allow such scientific evidence to be continually integrated at all micro-, meso-, and macro-levels of health care [15], improving both health and cost-effectiveness outcomes.

There is a scarcity of data in literature on healthcare resource utilization associated with mitochondrial diseases and the clinical drivers of these healthcare costs. A retrospective analysis of healthcare utilization for patients with mitochondrial disease in USA indicated that the top three cost-intensive areas were inpatient care, surgery and prescription drugs [16]. Due to the method of data collection and patient coding to identify patients with mitochondrial disease, it was likely that many patients with mitochondrial disease were not included and that not all the coded patients had the condition. A recent Canadian study retrospectively estimated the healthcare costs incurred by the participants before and after their first (index) hospitalization with an ICD code for mitochondrial disease [17], with the authors noting similar limitations in relation to patient coding as the USA study. Notably, there is no data investigating the healthcare costs for patients with clinically or genomically confirmed mitochondrial diseases in the out-patient setting where most of the clinical care for mitochondrial disease takes place.

To address this gap, we performed a cross-sectional retrospective study of hospital out-patient resource utilization and costs associated with outpatient care of patients with a confirmed diagnosis of mitochondrial disease. We also stratified groups depending on the results of their genetic testing to assess the impact of this intervention on outpatient healthcare costs.

## Methods

### Data source

For this cross-sectional observational study, we enrolled the participants over a period of three years (from September 2018 to August 2021) from the Mitochondrial Disease Clinic at Royal North Shore Hospital (RNSH) in NSW, which provides clinical care for patients with mitochondrial disease aged sixteen years and above. RNSH is the leading Mitochondrial Disease Clinic in Australia and accepts interstate referrals and provides expert advice to clinicians in other States and Territories. Consecutive patients were recruited for enrolment in the study if they either had a genetically confirmed diagnosis or met strict diagnostic clinical criteria [18] with muscle biopsy findings supportive of a definite diagnosis of mitochondrial disease.

### Standard protocol approvals, registrations, and patient consents

The research study was approved by the North Sydney Local Health District Human Research Ethics Committee (NSLHD-HREC Reference number: LNR/17/HAWKE/268) in accordance with National Health and Medical Research Council (NHMRC) National Statement [19] and NSW Health Policy Directive [20]. Written informed consent was obtained from all participants or guardians of the participants in the study.

### Data collection

We accessed the Hospital and Emergency Department datasets and performed a retrospective chart review. This involved accessing the electronic medical records (eMR) as well as patients’ charts to ensure a comprehensive assessment of the multi-specialty investigations as recorded by all clinicians and allied health service providers. We confirmed data collected on demographic variables, phenotype and symptom burden and utilization of out-patient healthcare services and resources including physician consultations via face-to-face or telephone interview.

### Attribution of costs

The costs of the consultations in the outpatient settings including diagnostic and surveillance investigations and day-only procedures were calculated using the listing of Government subsidized Medicare services available on Medicare Benefits Schedule (MBS) online (Table 1, Additional file 1). Costs were calculated for the baseline investigations and the follow-up investigations requested whilst providing follow-up care during the patient’s clinical course. The cost of genetic testing, muscle biopsy and blood tests such as serum lactate and pyruvate were not included in the analyses as these were standard across all study participants at baseline.

**Table 1:** Demographic and socioeconomic characteristics of participants

	Total	Group 1	Group 2	Group 3
Mean age (years)	53.7	41.4	54.1	61.5
*n (%)*				
< 40 years	24 (25.5)	20 (43.5)	5 (29.4)	4 (13.8)
> 40 years	70 (74.5)	26 (56.5)	12 (70.6)	25 (86.2)
*Gender n (%)*				
Male	34 (36.2)	15 (32.6)	4 (23.5)	12 (41.4)
Female	60 (63.8)	31 (67.4)	13 (76.5)	17 (58.6)
*Support level n (%)*				
Have a paid carer	22 (23.4)	8 (17.4)	8 (47.1)	6 (20.7)
Living with parents	10 (10.6)	5 (10.9)	4 (23.5)	1 (3.4)
*Education level n (%)*				
< Year 12	22 (23.4)	10 (21.7)	7 (41.2)	5 (17.2)
Year 12	9 (9.6)	4 (8.7)	0	5 (17.2)
Trade/Apprenticeship	10 (10.6)	5 (10.9)	2 (11.8)	3 (10.3)
Diploma/Certificate	22 (23.4)	11 (23.9)	4 (23.5)	6 (20.7)
Higher University Degree	29 (30.9)	16 (34.8)	4 (23.5)	9 (19.6)
Unknown	2 (2.1)			

### Analyses

We defined the healthcare resource utilization as the cohort’s use of out-patient healthcare services including hospital resources available through out-patient clinics and diagnostic centers and physician resources. To determine whether healthcare resource utilization was influenced by the results of genetic testing, we stratified participants into three different groups. The first group (designated as Group 1) consisted of participants with mtDNA mutations (i.e., mtDNA point mutations or deletions). The phenotypic spectrum displayed in this group included presentations such as MELAS syndrome (Mitochondrial Encephalopathy, Lactic Acidosis, and Stroke-like episodes) and LHON (Leber Hereditary Optic Neuropathy), as well as other non-syndromic presentations. Participants with nDNA mutations were placed together in the second group (designated as Group 2). The predominant phenotype for these participants was autosomal dominant (AD) and autosomal recessive (AR) chronic progressive external ophthalmoplegia (CPEO) or optic atrophy. Two participants had MLASA (Myopathy, Lactic Acidosis, and Sideroblastic Anaemia) caused by YARS2 mutations. The mtDNA and nDNA mutations in groups 1 and 2 are listed in Table 2, Additional file 1. Participants with clinical features consistent with clinically definite mitochondrial disease, supportive muscle biopsy findings but no genetic cause identified were allocated to the third group (designated as Group 3).

**Table 2:** Reported neurological and non-neurological symptoms

Symptoms	Group 1 [N = 46] n (%)	Group 2 [N = 17] n (%)	Group 3 [N = 29] n (%)
Neurological	43 (93.5)	16 (94.1)	27 (93.1)
Cardiac	17 (37.0)	5 (29.4)	14 (48.3)
Gastrointestinal	34 (73.9)	8 (47.1)	17 (58.6)
Ophthalmological	32 (69.6)	16 (94.1)	22 (75.9)
Respiratory	4 (8.7)	3 (17.6)	4 (13.8)
Hearing impairment	33 (71.7)	11 (64.7)	15 (51.7)
Sleep disturbance	32 (69.6)	15 (88.2)	21 (72.4)
Diabetes mellitus	21 (45.7)	2 (28.6)	7 (24.1)

The cost figures are presented here in their original currency (Australian Dollars or $AUD). The costs are per person per annum. The Analysis of Variance (ANOVA) was performed to determine the differences in healthcare resource utilization amongst the three groups. We conducted all statistical analyses using IBM SPSS Statistics version 27.

## Results

**Fig. 1:**
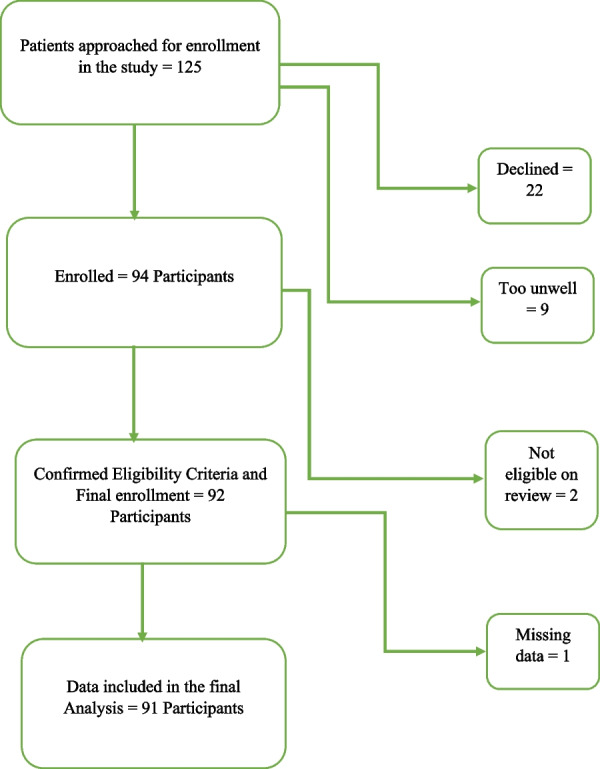
Participant enrollment and eligibility

We approached one hundred and twenty-five consecutive patients for enrollment in the study. Twenty-two patients declined to participate and nine were too unwell to participate, leaving ninety-four individuals who were recruited into the study. On further review, two participants did not meet the inclusion criteria and were excluded from the final analysis (Fig. 1). Overall, the response rate was 73.6%. The demographic and socioeconomic characteristics of the participants are summarized in Table 1.

The mean age of the participants at the time of recruitment was 53.72 years (range 16–83; SD 17.09) with 25.5% aged 16 to 40 years and 74.5% aged 41 to 83 years. Approximately two-thirds of the study participants were females (64%). The mean duration for which the participants had been attending the clinic was 9.12 years (range 1–19 years; SD 4.82).

The educational status of the study participants was variable. 30.9% had a higher university degree, 9.6% had completed higher school certificate (HSC) while 23.4% had not completed HSC. 10.6% of the participants had completed an apprenticeship after HSC and another 23.4% had a graduate diploma or certificate.

Neurological symptoms were the most commonly reported problem in our cohort as shown in Table 2. Table 2 also summarizes the frequency of the non-neurological symptoms reported in the three groups.

The most frequently reported non-neurological symptoms in Group 1 included gastrointestinal dysmotility (73.9%), hearing impairment (71.7%), ophthalmological problems (69.6%) and sleep disturbance and/or irregular sleep pattern (69.6%). In Group 2, ophthalmological problems were most prevalent (94.1%). These included reduced visual acuity (legal blindness in some), ptosis and problems associated with poor ocular motility secondary to CPEO. Sleep disturbance was frequently reported (88.2%) followed by hearing impairment (64.7%) and gastrointestinal dysmotility (47.1%). Ophthalmological problems were frequent in Group 3 (75.9%) followed by sleep disturbance (72.4%), gastrointestinal dysmotility (58.6%) and hearing impairment (51.7%). Cardiac symptoms were most prevalent in Group 3 (48.3%) and Diabetes Mellitus in Group 1 (45.7%). Non-sleep related respiratory symptoms were reported most frequently in Group 2 (17.6%) followed by Group 3 (13.8%) and then Group 1 (8.7%).

**Fig. 2:**
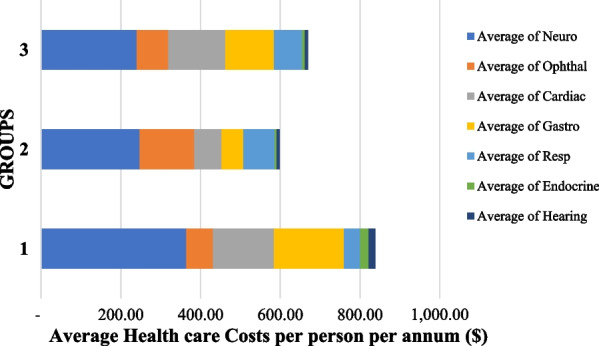
Total Average health care resource utilization and costs per person per annum

Figure 2 illustrates the composite of *the average out-patient costs per person per annum*. We found that patients with mtDNA encoded mitochondrial disease (Group 1) had the highest average out-patient costs per person per annum ($838.02; SD 809.72) followed by the patients without a confirmed genetic diagnosis (Group 3) which had the second highest average out-patient costs per person per annum ($669.12; SD 406.31). The annual average out-patient costs for nDNA encoded mitochondrial disease (Group 2) were $71 less ($598.12; SD 370.28) than that in Group 3. There was no significant difference in the average out-patient costs per person per annum (*p* = 0.34) between the three groups.

**Fig. 3:**
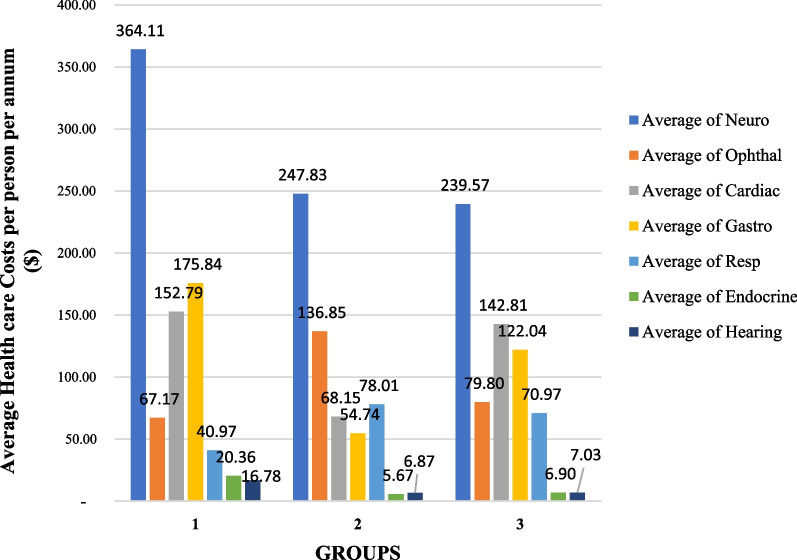
Average health care costs per person per annum by Specialty

We found that the neurological investigations were the largest driver of outpatient healthcare costs in all groups when compared to other investigations (Fig. 3). In Group 1, the average neurological cost per person per annum amounts to $364.11 (SD 340.93), in Group 2, to $247.83 (SD 113.86) and in Group 3, to $239.57 (SD 145.69). The difference in the neurological out-patient costs per person per annum amongst the three groups was not statistically significant (*p* = 0.09). Gastroenterological and cardiac related investigations and consultations were also major contributors to outpatient health care costs in Groups 1 and 3. In Group 1, the average gastroenterology-related costs per person per annum amounted to $175.84 (SD 300.06) and in Group 3, to $122.04 (SD 139.20) with no statistically significance difference between the two (*p* = 0.17). For cardiac investigations, cost contributions were $152.79 (SD 229.55) and $142.81 (SD 128.41) (*p* = 0.25) in Groups 1 and 3 respectively.

For patients with nDNA encoded mitochondrial disease (Group 2), following neurological investigations, ophthalmological ($136.85; SD 173.35), and then respiratory and cardiac investigations were the main drivers of out-patient costs for this group.

**Fig. 4:**
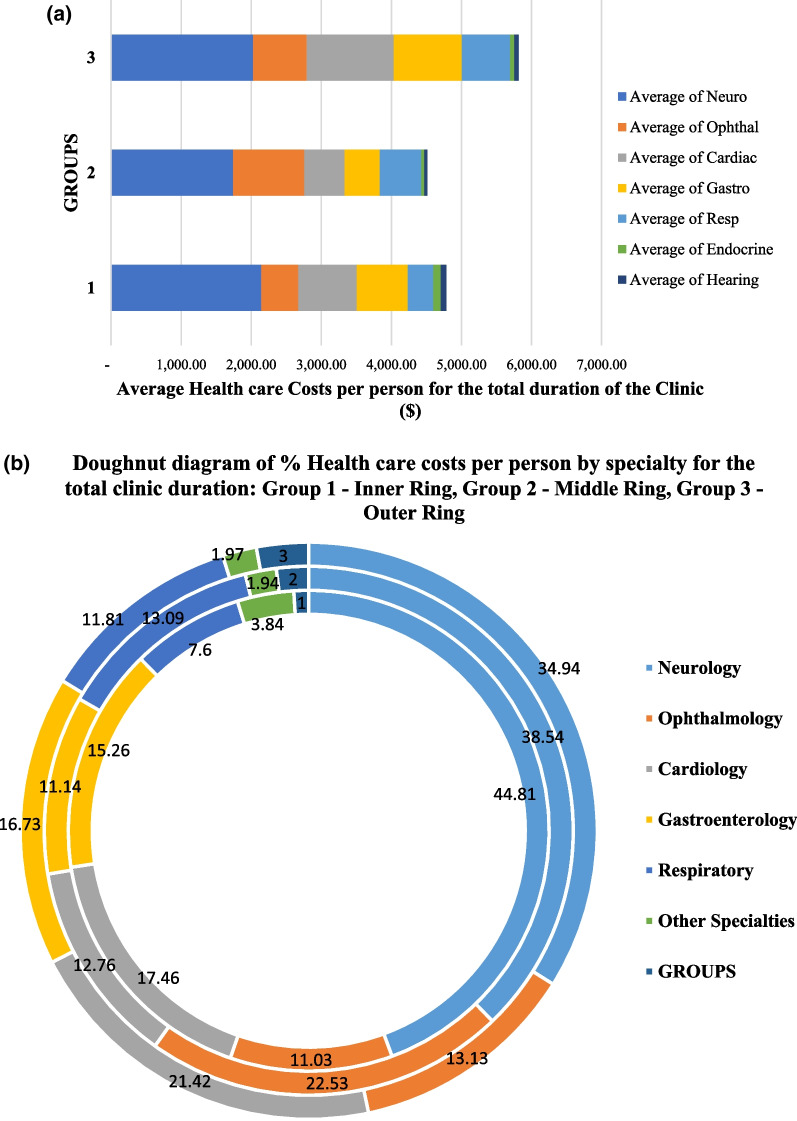
**a** Average health care resource utilization and costs per person for total duration of clinic attendance. **b** Percentage health resource utilization by specialty for the total duration of clinic attendance

When assessed *over the entire duration of the patients’ medical care* in the mitochondrial out-patient clinic, our study demonstrates that patients without a confirmed genetic diagnosis (Group 3) had the highest average healthcare resource utilization and costs per person. The average out-patient costs per person are illustrated in Fig. 4a. For Group 3, the out-patient costs per person amount to $5815.86 (SD 3520.40). This is followed by Group 1 at $4783.56 (SD 2859.11) and then Group 2 at $4514.84 (SD 2457.35).

For the total duration of medical out-patient care in the mitochondrial clinic, healthcare costs related to neurological investigations and consultations ranked the highest, contributing 44.81%, 38.54% and 34.94% respectively for groups 1, 2 and 3 (Fig. 4b). In group 2, ophthalmological investigations contributed 22.53%, ranking second highest. This was followed by respiratory and cardiac-related healthcare costs which contributed 13.09% and 12.76% of the out-patient costs respectively. In Groups 1 and 3, cardiac-related costs ranked second highest at 17.46% and 21.42% respectively. The gastroenterological investigations ranked third in both Groups 1 and 3 at 15.26% and 16.73% respectively.

## Discussion

Our study examines the clinical drivers of healthcare resource utilization in adult patients with mitochondrial disease in Australia in the out-patient setting.

Our results show that neurological consultations and investigations are the leading driver of healthcare costs in the out-patient clinics in all the three groups of participants: those with mtDNA mutations, those with nDNA mutations, and those with no genetic cause identified. This is consistent with the high frequency (94.5%) of neurological symptoms in our study population necessitating a higher frequency of neurological investigations and consultations. Patient symptoms requiring outpatient care included muscle fatigue and weakness, exercise intolerance, migraines, seizures, stroke-like episodes, cognitive deterioration, gait impairment, paraesthesia and sensory deficits and incoordination.

Those patients with mtDNA point mutations (Group 1) accrued the highest per person per annum neurological-driven out-patient costs compared to Groups 2 and 3 (Fig. 2). This is not surprising, given that neurological symptoms and signs contribute significantly to the clinical manifestations in the patients with mtDNA mutations [2–4]. In these patients, neurological investigations such as MRI brain with MR spectroscopy and electroencephalogram (EEG) were carried out more frequently due to the higher prevalence of certain neurological symptoms such as stroke-like episodes and seizures.

Despite the clinical heterogeneity and variability in their disease course, neurological complaints constituted a high symptom burden in the other two groups as well (Group 2: 94.1%; Group 3: 93.1%). Similarly, neurological investigation was the most resource intensive field in Groups 2 and 3 as well (Fig. 3). Neurological symptoms due to myopathy and peripheral neuropathy were frequently investigated and treated in patients with *OPA1* and *TWNK* mutations despite their features of optic atrophy, ptosis and ophthalmoplegia constituting the predominant phenotype [3, 21]. This higher frequency of neurological symptoms is similarly reflected in our study Group 3 which comprises participants with a clinical definite diagnosis but no confirmed genetic diagnosis to date, and is consistent with the study by Grier et al., where weakness, fatigue, difficulty in walking, incoordination, numbness and seizures were amongst the top ten self-reported symptoms in a cohort of patients with either a clinical diagnosis or biochemical deficiency or both [22].

Ophthalmological problems constituted the most frequent non-neurological symptoms (94.1%) and are consistent with outpatient monitoring of the ocular symptoms of participants stratified to Group2. Ophthalmological investigations and consultations are the second greatest driver of healthcare resource utilization. Ophthalmological costs were also attributed to day-only interventional procedures, of which surgery for correction of ptosis was most common. Surgical correction of ptosis in CPEO has shown safety and efficacy [23]. Eshaghi et al. have reported significant improvement in Margin-to-Reflex Distance 1 (MRD1) and chin-up posture at 1-year post-surgical follow up [24] and Doherty et al. demonstrated low complication rate and objective benefit following surgery in patients with ocular myopathy and ptosis [25]. In our cohort, 7 participants in Group 2 had ptosis correction surgery (41.2%) of which 3 patients required more than one operation. One participant in Group 1 and three participants in Group 3 also required ptosis correction surgery.

Gastroenterological and cardiac-related out-patient costs were also amongst the stronger drivers of healthcare resource utilization. Gastroenterological-related costs were the second most resource intensive field in Group 1 and third-most in Group 3. Almost three-quarters of the participants in Group 1 (73.9%) reported a high prevalence of gastrointestinal dysmotility and other symptoms associated with alimentary disorders. More than half of the participants in Group 3 (58.6%) also reported gastrointestinal symptoms. Similar to other studies [26–29], gastroenterological symptoms such as dysphagia, gastroesophageal reflux disease and feeding difficulties manifesting as delayed gastric emptying, constipation and intestinal pseudo-obstruction were observed and investigated with gastric motility and colonic transit studies and were main contributors to the higher out-patient costs. Gastroscopies and colonoscopies were performed at a lesser frequency. Consultations with gastroenterologists also contributed to the costs in this category especially for patients with severe or progressive symptoms.

Cardiac investigations and surveillance cardiac testing also contributed significantly to the healthcare resource utilization. The out-patient costs related to cardiac category are driven by surveillance investigations such as 12-lead electrocardiograms (ECGs), 24-h Holter monitoring and transthoracic echocardiograms (TTEs). Patients with mitochondrial disease are at an increased risk of cardiomyopathy and arrythmia as cardiac muscle is a high-energy-dependent tissue [30]. The progression to a high-grade AV block can be unpredictable in patients with mtDNA mutations, hence there is a need for regular monitoring despite minimal to no reported symptoms [31, 32]. Additionally, cardiac day procedures and investigations such as stress echocardiograms, electrophysiology studies (EPS) and myocardial perfusion scans (MIBI scans) are often more expensive compared to investigations in other specialties. Additional file 1: Table 1 lists the costs of specialty-based investigations and day-only procedures. Of the three groups, participants in Group 3 reported the highest prevalence of cardiac symptoms (48.3%), and investigations were more frequent because without a confirmed genetic diagnosis, the risks for cardiac complications are more difficult to ascertain.

When the health care costs were analyzed for the participants’ entire clinic duration, Group 3 showed the greatest healthcare resource utilization (Fig. 4). This observed difference is most likely due to the lack of a molecular diagnosis which can otherwise enable a more customized approach through utilization of streamlined standards of care and investigations [8, 9, 33] and undertaking further ancillary investigations to determine a potential molecular diagnosis.

## Strengths and limitations

The main strength of this study is that it is based on a sample of patients identified as having mitochondrial disease by a genetically confirmed diagnosis or clinically definite (with muscle biopsy positive) diagnosis of mitochondrial disease in a hospital out-patient setting when compared to other studies where there was uncertainty about whether the patients had a confirmed mitochondrial disorder [13].

One limitation of the study is underrepresentation of those patients with mitochondrial disease who were severely affected and deemed too unwell for recruitment. This may lead to under-estimation of health care resource utilization. Such patients require in-patient care more frequently. However, useful insights into the healthcare resource utilization and trends can be gained by analyzing the data from out-patient clinics prior to these patients requiring in-patient hospital care. A future study to estimate health care resource utilization for mitochondrial patients admitted to the hospital may help understand this gap further.

Children have a different disease pattern to adults with earlier age of onset, often greater disease severity and shorter disease duration. Our study focused on assessing the healthcare costs in the adult population, rather than a mixed adult and paediatric population given these differences. Hence, presentation and management in chronic condition outpatient departments is less. Studies investigating out-patient health care resource utilization in children with mitochondrial diseases may provide further insight into the breadth of the problem.

Reasons given by individuals who declined to enroll in the study included that they were either clinically stable or too busy managing daily routine and healthcare appointments and/or caring for additional family members. Individuals aged over 40 years who were not working full time were more amenable to recruitment. Similarly, there was greater participation by females compared to working male participants and this was independent of disease severity.

## Conclusion

Mitochondrial disease has protean clinical manifestations and individuals affected by this genetic disorder experience a complex multi-specialty clinic journey during their lifespan. The drivers of healthcare resource utilization and costs are dependent on the phenotype-genotype characteristics of the patients as well as the extent of the multisystem involvement, disease evolution and the rate of progression. The top three drivers for health care resource utilization and costs in the out-patient clinics are neurology, cardiology, and gastroenterology specialties unless the patient has nDNA mutations with predominant phenotype of CPEO and/or optic atrophy wherein ophthalmology is the second most resource intensive specialty after neurology.

## Supplementary Information

**Additional file 1: Table 1** Cost of investigations per specialty. **Table 2** Mutation details in groups 1 and 2.

## Data Availability

The authors have access to all the data in this study. The authors take full responsibility for the integrity of the data, the accuracy of the data analysis and interpretation and the conduct of the research. Due to data protection in accordance with approved ethical standards, the data cannot be made available publicly.

## Abbreviations

AD: Autosomal dominant
ANOVA: Analysis of variance
AR: Autosomal recessive
$AUD: Australian dollars
AV block: Atrioventricular block
CPEO: Chronic progressive external ophthalmoplegia
ECG: Electrocardiogram
EEG: Electroencephalogram
eMR: Electronic medical records
EPS: Electrophysiology study
HSC: Higher school certificate
ICD: International classification of diseases
LHON: Leber hereditary optic neuropathy
MBS: Medicare benefits schedule
MELAS: Mitochondrial encephalopathy, lactic acidosis, and stroke-like episodes
MIBI scan: Myocardial perfusion scan/sestamibi scan
MLASA: Myopathy, lactic acidosis, and sideroblastic anaemia
MRD1: Margin-to-reflex distance 1
MRI: Magnetic resonance imaging
mtDNA: Mitochondrial DNA
nDNA: Nuclear DNA
NHMRC: National Health and Medical Research Council
NSLHD-HREC: North Sydney Local Health District Human Research Ethics Committee
NSW: New South Wales
OPA1: Optic atrophy 1
RNSH: Royal North Shore Hospital
SD: Standard deviation
TTE: Transthoracic echocardiogram
TWNK: Twinkle mtDNA helicase
USA: United States of America
YARS2: Tyrosyl-tRNA synthetase 2
