# Supplementary material for: Healthcare resource utilization of patients with mitochondrial disease in an outpatient hospital setting
Source: Orphanet J Rare Dis. 2023 May 29;18:129. doi: 10.1186/s13023-023-02746-x (PMC10226231; doi:10.1186/s13023-023-02746-x)
Supplement: Supplementary file 1 — Additional file 1: Table 1 Cost of investigations per specialty. Table 2 Mutation details in groups 1 and 2. [file 13023_2023_2746_MOESM1_ESM.docx]

# **SUPPLEMENTARY MATERIAL**

**TABLE 1 - COST OF INVESTIGATIONS PER SPECIALTY**

| **NEUROLOGY** | |
| --- | --- |
| **Investigations / day-only procedures** | **Cost** |
| EEG (electroencephalogram) | 126.95 |
| NCS/EMG (nerve conduction study +/- electromyogram) | 230.95 |
| SFEMG (single fibre EMG) | 230.95 |
| BAER’s (Brainstem auditory evoked responses) | 198.5 |
| MRI – Brain +/- MRS (MR spectroscopy) | 403.2 |
| MRI whole spine | 358.4 |
| MRI cervical spine | 358.4 |
| MRI lumbosacral spine | 358.4 |
| VEPs (Visual evoked potentials) | 117.4 |
| MEPs (Motor evoked potentials) | 117.4 |
| Autonomic tests | 154.6 |
| Lumbar puncture – CSF studies | 169.85 |
| CT - Brain | 198 |
| CTA (angiogram) | 517.65 |
| CT paranasal sinuses | 228.35 |
| **OPHTHALMOLOGY** | |
| **Investigations / day-only procedures** | **Cost** |
| OCT (optical coherence tomography) | 41.25 |
| VF (visual fields) bilateral | 69.9 |
| VF (visual fields) unilateral | 42.1 |
| Ptosis surgery | 966.70 |
| Strabismus surgery | 604.85 |
| X-ray orbits/facial bones/sinuses | 48 |
| ERG (electroretinogram) | 111.65 |
| **GASTROENTEROLOGY** | |
| **Investigations / day-only procedures** | **Cost** |
| Abdominal X-ray (AXR) | 36.25 |
| Abdomen US (ultrasound) | 38.4 |
| CT abdomen | 568.4 |
| Ba (barium) Swallow | 91.3 |
| Bowel Transit studies | 687.7 |
| Gastric emptying study | 574.35 |
| Colonoscopy | 344.8 |
| Gastroscopy | 182.65 |
| MR enterography | 457.2 |
| Anal manometry | 192.65 |
| **CARDIOLOGY** | |
| **Investigations / day-only procedures** | **Cost** |
| ECG (electrocardiogram) | 32.25 |
| TTE (trans-thoracic echocardiogram) | 234.15 |
| Trans-oesophageal echocardiogram (TOE) | 279.65 |
| 24-hour Holter monitor | 172.75 |
| Stress exercise Test/Echo | 417.45 |
| PPM insertion | 1126.95 |
| AICD insertion | 1085.55 |
| EPS (electrophysiology study) | 850.95 |
| Ablation | 1415.3 |
| Tilt table testing | 174.15 |
| CTCA (CT coronary angiogram) | 710.5 |
| Myocardial perfusion scan | 653.05 |
| Venous Doppler US | 172.05 |
| Ambulatory BP monitoring | 95 |
| PCA (Percutaneous Coronary Angiogram) | 548.85 |

**TABLE 2 - MUTATION DETAILS IN GROUPS 1 AND 2**

| **GROUP 1 (mtDNA point mutations and deletions)** | | |
| --- | --- | --- |
| **MUTATIONS** | **n** | **Total number of participants (N)** |
| m. 3243 A>G | 33 | **46** |
| m. 3251 A>G | 2 |  |
| m. 3256 C>T | 1 |  |
| m. 4269 A>G | 1 |  |
| m. 8344 A>G | 1 |  |
| m. 990 T>G | 1 |  |
| m. 11778 G>A | 1 |  |
| m. 13042 G>A | 1 |  |
| m. 13528 A>G | 1 |  |
| m. 14674 T>C | 1 |  |
| mDNA deletions | 3 |  |
| **GROUP 2 (nDNA mutations)** | | |
| **MUTATIONS** | **n** | **Total number of participants (N)** |
| C10Orf2/TWNK | 3 | **17** |
| OPA1 | 8 |  |
| POLG | 4 |  |
| YARS2 | 2 |  |
